# The therapeutic significance of the novel photodynamic material TPE-IQ-2O in tumors

**DOI:** 10.18632/aging.202355

**Published:** 2020-12-19

**Authors:** Liyu Wang, Yu Liu, Hengchang Liu, He Tian, Yalong Wang, Guochao Zhang, Yuanyuan Lei, Liyan Xue, Bo Zheng, Tao Fan, Yujia Zheng, Fengwei Tan, Qi Xue, Shugeng Gao, Chunxiang Li, Jie He

**Affiliations:** 1 Department of Thoracic Surgery, National Cancer Center, National Clinical Research Center for Cancer, Cancer Hospital, Chinese Academy of Medical Sciences and Peking Union Medical College, Beijing 100021, China; 2 Department of Colorectal Surgery, National Cancer Center, National Clinical Research Center for Cancer, Cancer Hospital, Chinese Academy of Medical Sciences and Peking Union Medical College, Beijing 100021, China; 3 Department of Pathology, National Cancer Center, National Clinical Research Center for Cancer, Cancer Hospital, Chinese Academy of Medical Sciences and Peking Union Medical College, Beijing 100021, China

**Keywords:** photodynamic therapy, immunotherapy, surgery, combination therapy, tumor microenvironment

## Abstract

Combination therapies based on photodynamic therapy (PDT) have received much attention in various cancers due to their strong therapeutic effects. Here, we aimed to explore the safety and effectiveness of a new mitochondria-targeting photodynamic material, TPE-IQ-2O, in combination therapies (combined with surgery or immunotherapy). The safety and effectiveness of TPE-IQ-2O PDT were verified with cytotoxicity evaluation *in vitro* and a zebrafish xenograft model *in vivo*, respectively. The effectiveness of TPE-IQ-2O PDT combined with surgery or immune checkpoint inhibitors (ICIs) was verified in tumor-bearing mice. Small animal *in vivo* imaging, immunohistochemistry, and flow cytometry were used to determine the underlying mechanism. TPE-IQ-2O PDT can not only reduce tumor recurrence in surgical treatment but also effectively improve the response to ICIs in immunotherapy without obvious toxicity. It was also found to ameliorate the immunosuppressive tumor microenvironment and promote the antitumor immunity induced by ICIs by increasing CD8^+^ tumor-infiltrating lymphocyte accumulation. Thus, TPE-IQ-2O PDT is a safe and effective antitumor therapy that can be combined with surgery or immunotherapy.

## INTRODUCTION

Photodynamic therapy (PDT) is a novel minimally invasive therapy for tumors with relatively high specificity. Photosensitizer (PS), oxygen and light are the three elements of PDT [1, 2]. PDT utilizes a PS activated by specific wavelengths of light under aerobic environments to produce reactive oxygen species (ROS). ROS can induce cancer cell death directly or indirectly by causing damage to the tumor vasculature. Traditional PDT is effective in treating superficial cancers, such as bladder cancer [3]. Numerous PSs have been developed to increase tumor targeting. Especially, some PSs based on subcellular organelles targeting has been developed recently and witnessed some exciting results [4]. Unfortunately, given the hypoxic tumor microenvironment and limited penetration depth of light, a single PDT modality used alone usually shows poor therapeutic efficacy *in vivo* [5–7]. Although PDT cannot completely eliminate solid tumors, the cytotoxicity of PDT may greatly promote tumor antigen release, initiating a cascade of immune responses [8]. Recent evidence suggests that immune checkpoint inhibitors (ICIs) and PDT may complement each other in the antitumor process [9].

Programmed cell death ligand 1 (PD-L1) and programmed death 1 (PD-1) are mainly expressed on the membrane surface of various tumor cells and CD8+ T cells, respectively. PD-1 and its ligand PD-L1 are key coinhibitory molecules in tumor evasion and are also the most valuable targets exploited in cancer immunotherapy [10, 11]. ICIs targeting the PD-1/PD-L1 pathway have achieved satisfactory results in the treatment of melanoma, classic Hodgkin lymphoma and some solid tumors [12–14]. However, ICI therapy exhibits limited effects in some cancer patients, which might be attributed to the immunosuppressive state in these patients [15–17]. Combining chemotherapy or radiotherapy with ICIs has been developed to overcome the insensitivity to PD-1/PD-L1 ICIs in these patients [18]. Thus far, the response rates of PD-1/PD-L1 ICIs combined with other therapies are inconsistent. Some studies have shown that combination therapy, such as an anti-PD-L1 monoclonal antibody combined with docetaxel, is no better than immunotherapy alone [19–22]. The reason for this difference is unclear but may lie in the side effects and poor targeting of chemotherapeutics. Due to its high specificity and immunomodulatory effects, PDT is a promising strategy for achieving synergistic anticancer activities with immunotherapy or other therapies [9, 23–25].

TPE-IQ-2O is a new kind of aggregation-induced emission (AIE) material. Compared with common aggregation-caused quenching (ACQ) materials, such as fluorescein isothiocyanate (FITC), AIE materials display high biocompatibility, excellent photostability and strong resistance to photobleaching. An increasing number of AIE material studies are now receiving widespread attention in antitumor applications, but TPE-IQ-2O has been reported extensively in the literature. Gui C and Tang BZ et al reported that TPE-IQ-2O served as an ideal PS and could specifically target the mitochondria of tumor cells [26–28]. Regrettably, *in vivo* experiments and the value of combination therapy have not been investigated. The purpose of this research was to evaluate the efficacy of TPE-IQ-2O PDT combined with surgery or a PD-L1 inhibitor *in vivo*. This investigation utilized various tumor cell lines and a tumor-bearing animal model to assess the biological safety and efficacy of TPE-IQ-2O PDT as well as the value of TPE-IQ-2O-related combination therapy.

## RESULTS

### TPE-IQ-2O specifically targets mitochondria

**Figure 1:**
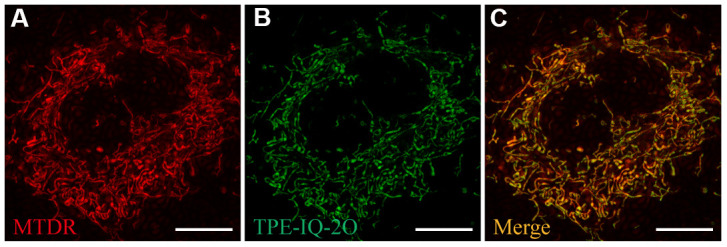
**Ultra-high-resolution fluorescence imaging of A549 cells.** (**A**) MTDR staining (50 nM). (**B**) TPE-IQ-2O (200 nM) staining. (**C**) Merged image of panels (**A**) and (**B**). λ_ex_: 650 nm (MTDR) and 488 nm (TPE-IQ-2O); scale bar =10 μm.

First, A549 cells were co-stained with TPE-IQ-2O (200 nM) and MTDR (50 nM) for 20 minutes to confirm the intracellular localization of TPE-IQ-2O. MTDR is a special dye that targets the mitochondria. The fluorescence signals from MTDR and TPE-IQ-2O were observed (Figure 1A, 1B). The overlap coefficient and correlation coefficient for the two images were calculated to be 0.843 and 0.846, respectively (Supplementary Figure 2). The results showed that TPE-IQ-2O was specifically located in the mitochondria (Figure 1C).

### TPE-IQ-2O PDT targets tumor cells and promotes apoptosis

**Figure 2:**
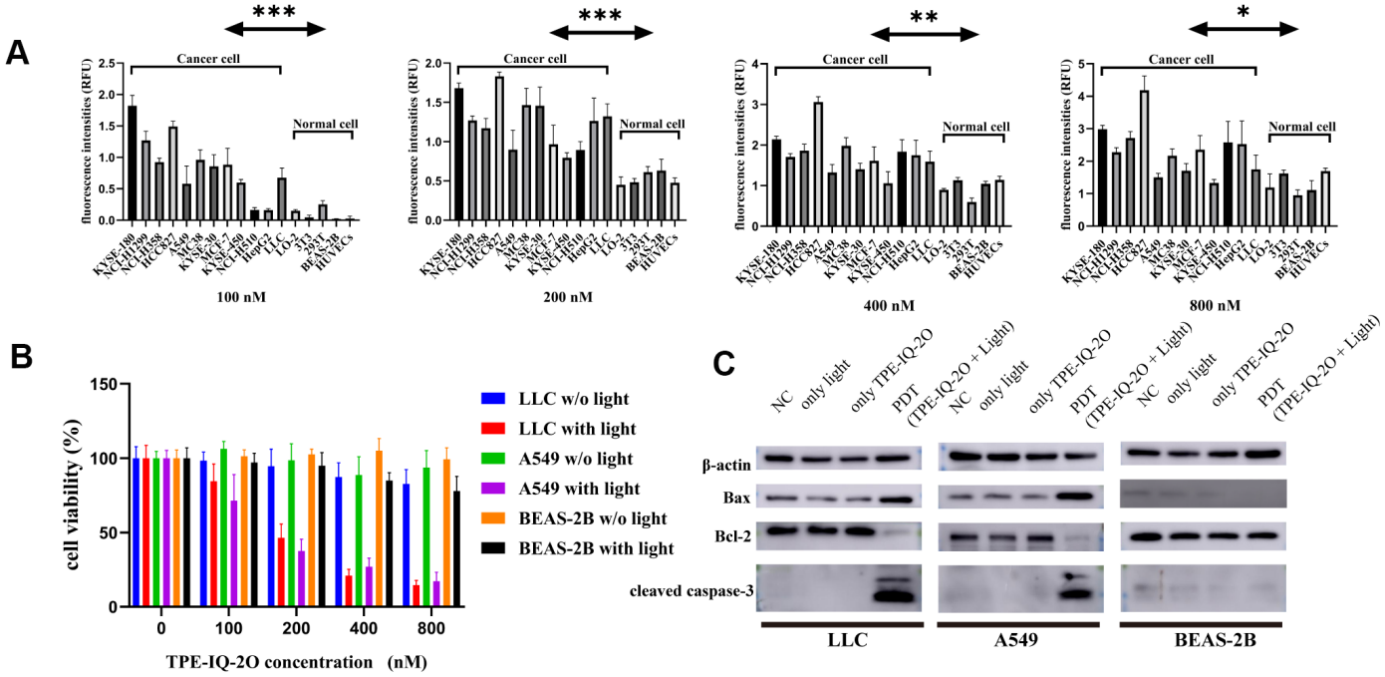
**TPE-IQ-2O PDT targets tumor cells and promotes apoptosis.** (**A**) The fluorescence intensities of tumor cells and normal cells incubated with different concentrations of TPE-IQ-2O were measured; λ_ex_: 430 nm, λ_em_: 560 nm, *P<0.05, **P<0.01, ***P<0.001 by unpaired Student’s t-test. (**B**) TPE-IQ-2O with white light (5 W, 85 mW/cm^2^) irradiation killed stained LLC and A549 cells but had no significant effect on the viability of BEAS-2B cells, w/o represents the abbreviation of without in this figure. (**C**) The protein expression of Bax, Bcl-2 and cleaved caspase-3 was evaluated.

Next, to determine whether TPE-IQ-2O specifically targets tumor cells, multiple tumor and normal cell lines were treated with gradient concentrations of TPE-IQ-2O (100/200/400/800 nM). The fluorescence intensities of all stained cell lines were further measured with an enzyme labeling instrument. After treatment with TPE-IQ-2O, all tumor cells showed a higher fluorescence signal than normal cells. In Figure 2A, it seemed that both 100 nM and 200 nM were suitable concentration to differentiate normal cells and tumor cells (p<0.001). However, we found that two cancer cell lines (NCI-H510 and HepG2) showed lower signals than one normal cell line (293T) at the concentrations of 100 nM. By comparison, all cancer cell lines displayed higher signals than normal cell lines at the concentrations of 200 nM (Supplementary Figure 3A). In addition, we have detected cell viabilities by CCK8 assay at the concentrations of 100 nM and 200 nM, respectively. The results of the CCK8 assay further verified that 200 nM was the effective concentration resulting in about 50% inhibition of cancer cells *in vitro* without obvious toxicity to normal cells. Cell viability analysis of normal cells revealed no significant difference between the concentrations of 100 nM and 200 nM (Supplementary Figure 3A). Therefore, we suggested that 200 nM as the optimal concentration for TPE-IQ-2O.

It was obvious that the staining rate of tumor cells was higher than that of normal cells, indicating that TPE-IQ-2O may predominantly target tumor cells (Figure 2A). Previous research suggested that TPE-IQ-2O accumulates in tumor cells based on higher MMP than normal cells (Supplementary Figure 4A) [24]. Further investigation was warranted to find out whether the different targetability of TPE-IQ-2O is correlated with MMP. The ratio of red/green fluorescence of JC-1 can reflect the MMP of cells. KYSE-30, LLC, A549 and BEAS-2B cells were stained with JC-1. Subsequently, JC-1 signals were analyzed by flow cytometry. By observing the red/green ratio, the fluorescent images (Supplementary Figure 4B) were consistent with the flow cytometry result (Supplementary Figure 4C). According to Supplementary Figure 4C, while BEAS-2B cells showed the lowest MMP, KYSE-30 cells possessed the highest value and the trend of TPE-IQ-2O fluorescence intensity paralleled that of MMP level (the red/green ratio). These results indicate that TPE-IQ-2O can distinguish tumor cells from normal cells by MMP.

We further analyzed cell proliferation in LLC (mouse lung cancer cell line), A549 (human lung cancer cell line) and BEAS-2B (normal cell line) cells treated with TPE-IQ-2O PDT. CCK-8 data demonstrated that TPE-IQ-2O with light exposure (5 W, 85 mW/cm^2^) could inhibit LLC and A549 cell proliferation *in vitro* in a dose-dependent manner. However, there were no obvious effects of other treatments on either the BEAS-2B, LLC or A549 cell line (Figure 2B). Additionally, apoptosis-related proteins, including cleaved caspase-3, Bcl-2 and Bax, were detected in the LLC, A549 and BEAS-2B cell lines. As expected, cleaved caspase-3 and Bax/Bcl-2 expression was significantly higher in the TPE-IQ-2O PDT group than in the control group for the LLC and A549 cell lines. In contrast, there was no significant different between the groups with BEAS-2B cells (Figure 2C). The results show that TPE-IQ-2O PDT can inhibit the viability of tumor cells and induce tumor cell apoptosis.

### TPE-IQ-2O PDT has an ideal effect *in vivo*

**Figure 3:**
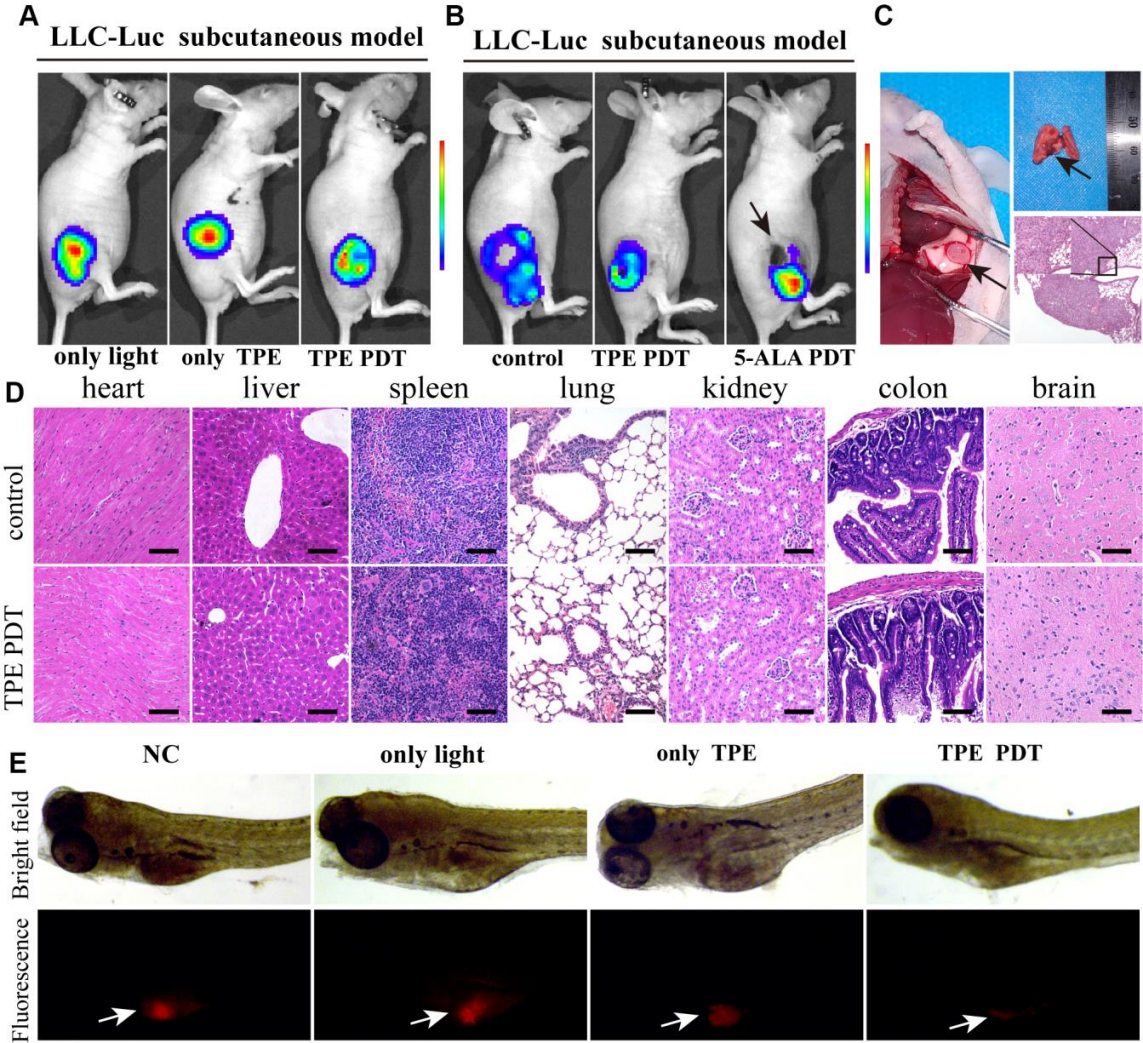
**TPE-IQ-2O has a relatively strong PDT effect *in vitro*.** (**A**) The killing effect of TPE-IQ-2O as a photosensitizer on tumor tissue requires exposure to light. (**B**) *In vivo* small animal imaging was performed on day 7 after TPE-IQ-2O PDT or traditional 5-ALA PDT. The black arrow shows burned skin and tumor festering. (**C**) The anatomical findings for metastatic lung cancer after 5-ALA PDT (Day 14, indicated by the black arrow) and pathological HE staining are shown. (**D**) Representative images of HE staining of tissue samples from the TPE-IQ-20 PDT group and control group are shown, scale bar = 50 μm. (**E**) TPE-IQ-2O was performed with/without white light (5 W, 85 mW/cm^2^) irradiation in a zebrafish mCherry^+^ A549 cell xenograft model. (TPE represents the abbreviation of TPE-IQ-2O in this figure.)

Then, the LLC-Luc cell line was used to establish tumor-bearing nude mice, which were treated with white light, TPE-IQ-2O or both. TPE-IQ-2O PDT exhibited a stronger antitumor effect at six hours after treatment than light or TPE-IQ-2O monotherapy (Figure 3A, Supplementary Figure 5A). 5-ALA is a commonly used PDT drug in the clinic with definite effects. We attempted to compare the advantages and disadvantages of the two materials (5-ALA and TPE-IQ-2O) in PDT. TPE-IQ-2O and 5-ALA were used independently as PSs to treat subcutaneous tumors in tumor-bearing mice. Seven days later, the volumes of the subcutaneous tumors in the mice treated with each of the two photodynamic drugs were significantly smaller than that in control mice. The 5-ALA PDT group had obvious necrotic foci at the irradiation point with strong tumor growth activity in the surroundings. In addition, the tumor activity in the TPE-IQ-2O PDT group was lower than that in the 5-ALA PDT group (Figure 3B, Supplementary Figure 5B). Subsequently, all mice were dissected for histological evaluation. There were two cases of pulmonary metastasis in the 5-ALA PDT group, but no distant metastases were found in the control group or TPE-IQ-2O PDT group (Figure 3C). We then quantified Dihydroethidium (DHE), a ROS probe, to compare the extracellular ROS generation capability of TPE-IQ-2O and 5-ALA. We found that TPE-IQ-2O and 5-ALA did not show significant differences in their ability of changing ROS level in cancer cells. However, 5-ALA produced significant amount of ROS in normal cells (*P*<0.001) (Supplementary Figure 6). This result nicely corroborates the conclusion that the TPE-IQ-2O PDT was capable of targeting tumor cells without obvious damage on normal cells. Furthermore, HE staining showed that there were no significant changes in pathological structures in the main organs of mice between the control group and the TPE-IQ-2O PDT group (Figure 3D). We also added the comparison of biochemical and blood cell analysis in control and TPE-IQ-2O PDT group (Supplementary Figure 7). There was no significant difference in above parameters between the two groups. Thus, TPE-IQ-2O PDT showed satisfactory performance in bio-safety.

Since zebrafish are transparent, white light penetrates them better than mice. Therefore, zebrafish model can eliminate the factor of insufficient light penetration when estimating the photodynamic effect *in vivo*. In a zebrafish xenograft assay, red fluorescence represented live tumor cells, namely, mCherry^+^ A549 cells. In the TPE-IQ-2O PDT group, tumors were thoroughly killed. All zebrafish in all groups survived during the experimental period. This result demonstrates the efficiency of TPE-IQ-2O PDT (Figure 3E).

### TPE-IQ-2O PDT combined with surgery can reduce tumor recurrence

**Figure 4:**
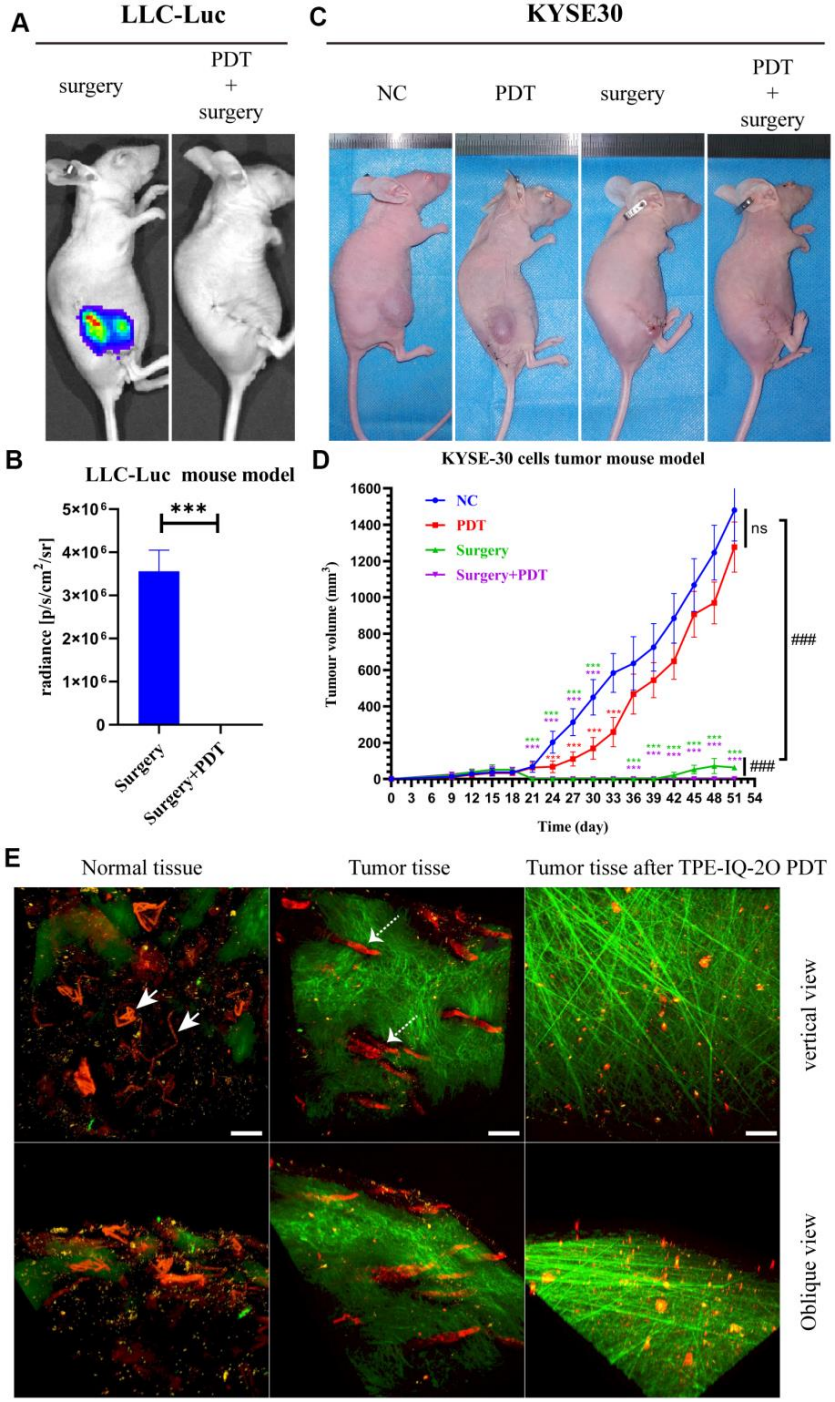
**TPE-IQ-2O PDT combined with surgery can effectively remove the residual tumor focus at the incisal margin and reduce tumor recurrence.** (**A, B**) *In vivo* bioluminescence imaging and measurement of the LLC-Luc cell tumor mouse model after surgery alone or combined with intraoperative PDT therapy on the 17^th^ day. n=3 mice/group. Data are expressed as the mean±s.d., ***p < 0.001 (**C**, **D**) A KYSE-30 cell tumor mouse model and tumor volumes in the control group, PDT alone group, surgery alone group and surgery combined with intraoperative PDT group. n=5 mice/group. Data are expressed as the mean±s.d., ***p < 0.001 vs. control group following the Dunnett or Dunnett’s T3 test, ###*p* < 0.001 for pairwise comparison on day 51 following Dunnett’s T3 post hoc test. (**E**) Evaluation of tumor vessels and the stroma after TPE-IQ-2O PDT in the KYSE30 cell subcutaneous tumor model (the white solid arrow shows subcutaneous vessels in normal skin, and the white dashed arrow shows tumor vessels; λ_ex_: 1040 nm, λ_em_: 575-610 nm; the green signal represents the stroma, λ_em_: 805 nm; the red signal represents vessels, scale bar=20 μm). (PDT represents the abbreviation of TPE-IQ-2O PDT in this figure).

Nude mice bearing LLC-Luc cells were used to evaluate the value of TPE-IQ-2O PDT in the context of surgical procedures, respectively. The results of LLC-Luc xenograft model showed that tumor recurrence occurred more frequently in the surgery alone group than in the PDT combined with surgery group. In the surgery group, all 5 mice had local recurrences, and the mean recurrence time was 12.6 days (SD 3.8 days; range 8-17 days). In contrast, recurrence was not reported in the PDT combined with surgery group in the following 8 weeks of observation (Figure 4A, 4B).

The above treatments were repeated with BALB/c nude mice bearing KYSE-30 cancer cells. On the 18^th^ day after subcutaneous tumor formation, we surgically removed tumors. Recurrent tumors could be seen in the surgical margin on postoperative day 21 (the 39^th^ day after subcutaneous tumor formation) in the surgery group. As shown in Figure 4C, 4D, there were obvious antitumor effects in the PDT combined with surgery group (*P*<0.001). The PDT group showed obvious effectiveness in the early stage (from day 24 to day 33, *P*<0.001) and a lesser effect in the later stage (from day 36 to day 51). Additionally, differences in the tumor vasculature and peritumoral stroma were observed by two-photon fluorescence microscopy. As expected, blood vessels in the tumor mass were destroyed after TPE-IQ-2O PDT. Additionally, the peritumoral stroma underwent remarkable morphological changes and displayed a loose cross-arrangement of fibers in the TPE-IQ-2O PDT group (Figure 4E).

**Figure 5:**
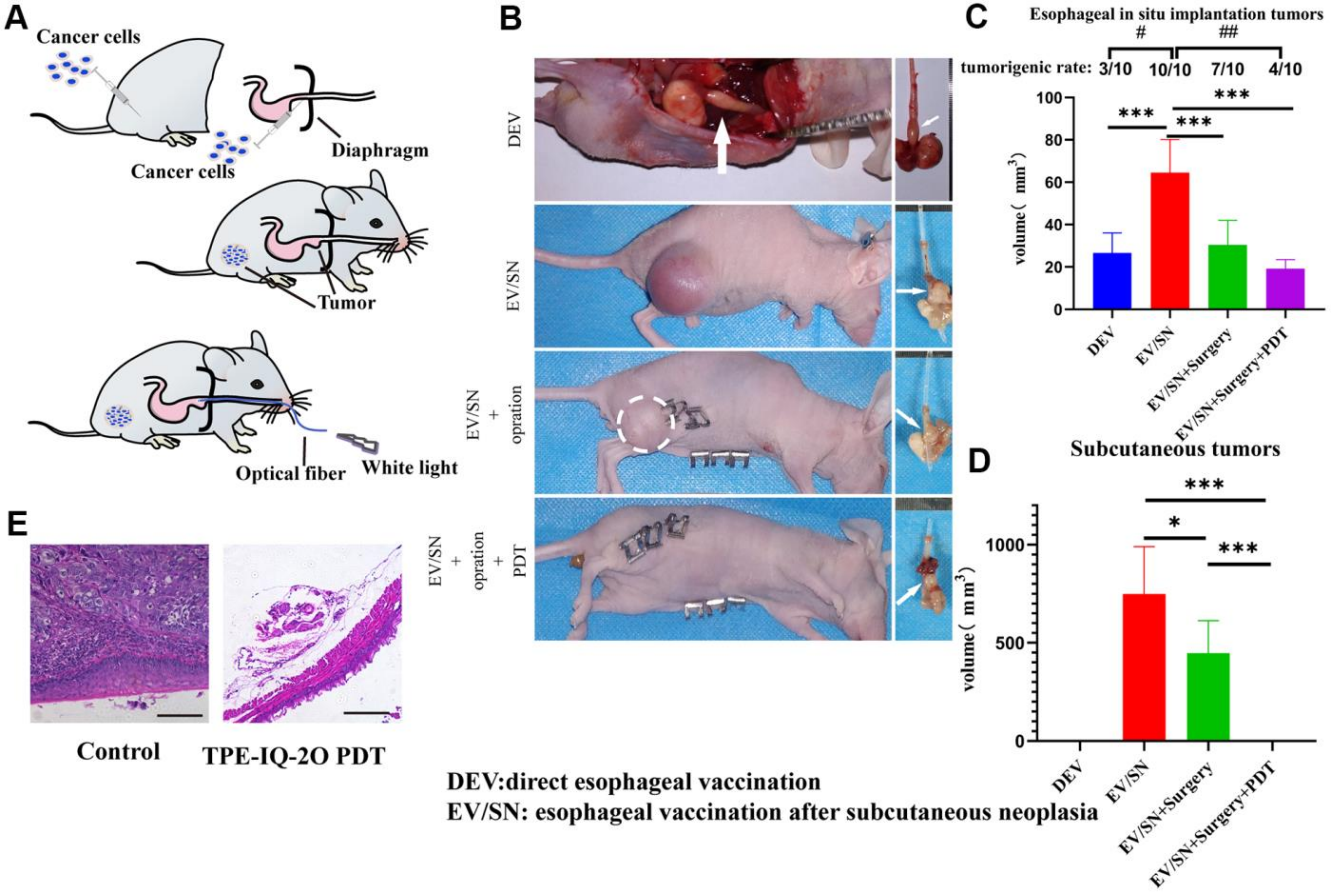
**TPE-IQ-2O PDT combined with surgery can reduce the tumor formation rate, size of orthotopically implanted esophageal carcinoma and primary tumor recurrence of subcutaneous tumors.** (**A**) Schematic diagram of subcutaneous tumorigenesis, esophageal orthotopic tumor implantation and transesophageal PDT in nude mice. (**B**) Gross dissection specimens of the DEV group, EV/SV group, EV/SV surgery group, and EV/SV surgery combined with PDT group (the white circle indicates the recurrent focus at the incision, and the white arrow shows the esophageal tumor *in situ*). (**C**, **D**) Volumes of the subcutaneous tumor and implanted esophageal tumor in each group (volume = 1/2 long diameter×short diameter^2^; B, C and (**D**) show data for day 14 after tumor reduction with PDT administered every three days for 15 minutes each time, #*P* < 0.05 and ##*P* < 0.01 for pairwise comparison following Fishers exact Chi-square test, *P < 0.05 and ***P < 0.001 for pairwise comparison following Dunnett’s T3 post hoc test). (**E**) HE staining of implanted orthotopic esophageal tumors treated with or without TPE-IQ-2O PDT.

In addition, an orthotopic implantation tumor model and subcutaneous xenograft model were established with KYSE-30 cells to evaluate whether local TPE-IQ-2O PDT had any effect (abscopal effect) on distant tumors (Figure 5A). The abbreviation DEV is used to refer to the model in which KYSE-30 cells were directly injected into the mouse esophageal mucosa. The term ‘EV/SN’ is used to describe the model in which KYSE-30 cells were injected into the esophageal mucosa after subcutaneous tumor formation. Compared with the EV/SN group, the EV/SN +surgery group and the EV/SN +surgery+ PDT group showed more significant tumor growth inhibition in the context of orthotopic transplantation (*P*<0.001) (Figure 5B, 5C). Both groups also showed significantly reduced recurrence in the distant subcutaneous incision. However, the rate of tumorigenesis in EV/SN + surgery + PDT group (4/10) was significantly lower than that in the EV/SN + surgery group (10/10) (*P*<0.01) (Figure 5C, 5D). Additionally, compared with the EV/SN group, the EV/SN +surgery + PDT group showed that direct irradiation did not damage esophageal mucosal integrity, as determined by HE staining (Figure 5E).

### TPE-IQ-2O PDT can effectively improve the response to BMS202

**Figure 6:**
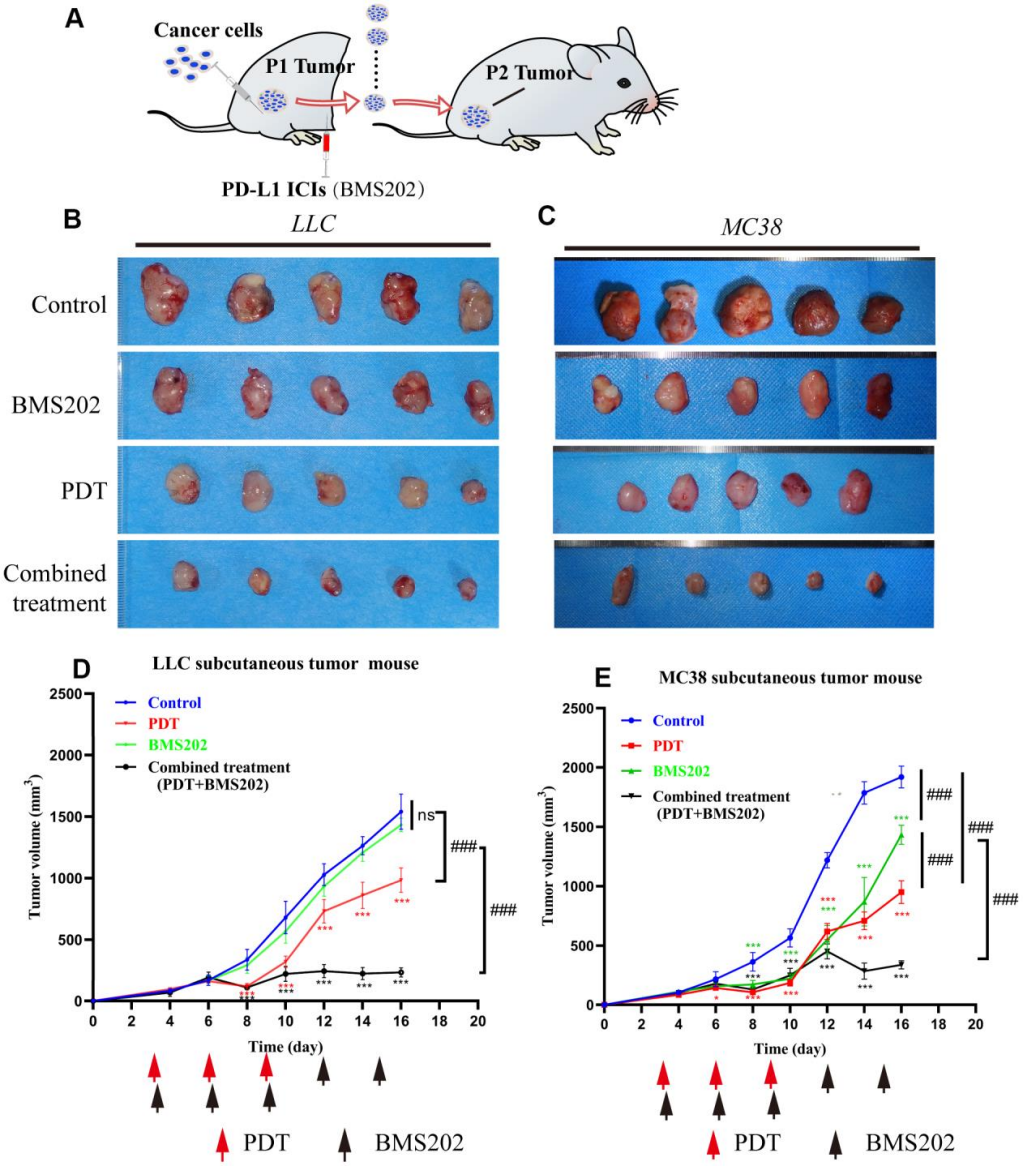
**TPE-IQ-2O PDT combined with a PDL1 ICI can effectively inhibit tumor growth.** (**A**) Schematic diagram of the establishment of a relatively ICI-insensitive animal model. (**B**, **C**) The tumors in the LLC or MC38 tumor-bearing mice of the control group (n=5), TPE-IQ-2O PDT group (n=5), BMS202 group (n=5) and combined treatment (PDT combined with BMS202) group (n=5). (**D**, **E**) Tumor growth curves for LLC or MC38 tumor-bearing mice, *p < 0.05,***p < 0.001 vs. control group following the Dunnett test, ###p < 0.001 for pairwise comparison on day 16 following LSD test).

Tumor-bearing C57/B6 mouse models were established with LLC and MC38 cells to evaluate the effect of TPE-IQ-2O PDT in combination with immunotherapy (Figure 6A). We created four groups with different treatments including the control group, the PDT group, the BMS202 (a PD-L1 ICI) group and the combined treatment (TPE-IQ-2O PDT + BMS202) group. For the BMS202 group, BMS202 was injected intraperitoneally every 3 days beginning on the 3^rd^ day after tumor formation. Similarly, PDT was performed on the 3^rd^, 6^th^, 9^th^, 12^th^ and 15^th^ days in the TPE-IQ-2O PDT+ BMS202 group.

In LLC tumor-bearing C57/B6 mice, tumor tissue growth was inhibited significantly in the PDT treatment alone group and TPE-IQ-2O PDT + BMS202 group (*P*<0.001). In addition, TPE-IQ-2O PDT + BMS202 had a significantly stronger effect than PDT treatment alone (*P*<0.001). However, BMS202 alone was unable to suppress tumor growth (Figure 6B, 6D, *P*>0.05). In the MC38 tumor-bearing C57/B6 mouse model, we found that the TPE-IQ-2O PDT group and the TPE-IQ-2O PDT + BMS202 group exhibited obvious antitumor effects. The tumor volume of the BMS202 group did not significantly increase during the early stage, revealing a growth trend similar to that of TPE-IQ-2O PDT group and the TPE-IQ-2O PDT + BMS202 group. However, the BMS202 group quickly recovered and showed a rapid rate of increase on the 10^th^ day and the difference was significant compared with the two remaining groups (Figure 6C, 6E, *P*<0.001).

### TPE-IQ-2O PDT promotes BMS202 antitumor immunity by increasing CD8^+^ tumor-infiltrating lymphocyte accumulation

**Figure 7:**
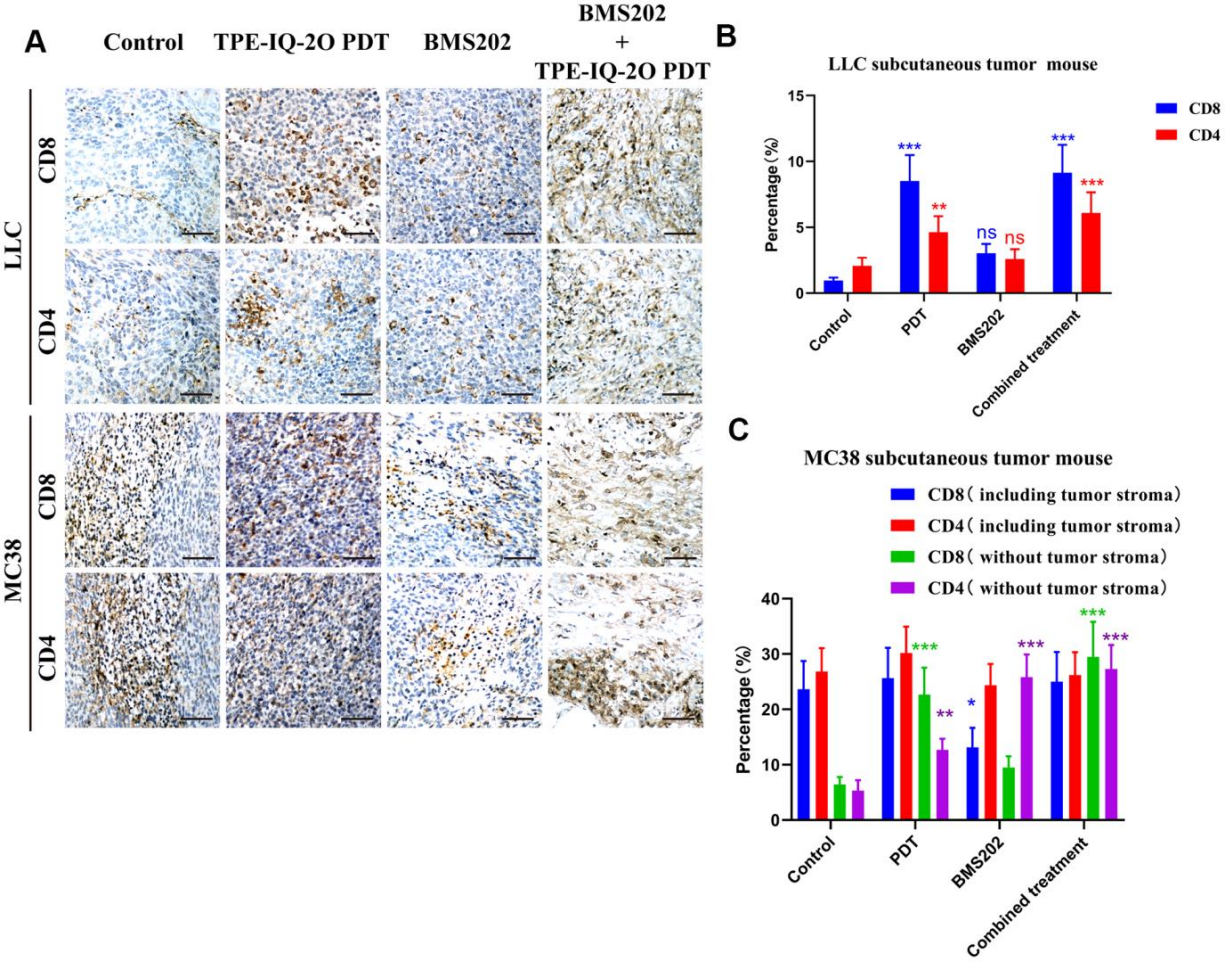
**Immunohistochemistry showed that TPE-IQ-2O PDT combined with immunotherapy increased the infiltration of CD8^+^ T cells into tumors.** (**A**) Microscopic observation of CD4+CD8+ T lymphocyte infiltration in the subcutaneous LLC or MC38 tumors in each group, scale bar: 150 μm. (**B**, **C**) The proportion of CD4+CD8+ T lymphocytes in the subcutaneous LLC or MC38 tumors in each group; *P < 0.05, **P < 0.01 and ***P < 0.001 vs. control group).

Tumor-infiltrating lymphocytes (TILs), especially CD8^+^ subpopulations, are known to be significantly associated with the outcome of immunotherapy in cancers. [10] IHC assays were performed to detect the possible mechanism. We found that CD8^+^ and CD4 TILs were significantly increased in the PDT group and the PDT + BMS202 group in both LLC and MC 38 tumor tissue samples (Figure 7A).

Due to the lack of interstitial components in LLC tumors, we only calculated the proportion of lymphocytes to tumor cells. In the LLC subcutaneous tumor model, the proportions of CD8^+^ TILs in the control group, the PDT group, the BMS202 group and the combined treatment group were 0.960±0.221%, 8.523±1.963%, 3.044±0.701% and 9.146±2.106%, respectively, and the proportions of CD4^+^ infiltrating lymphocytes in four groups were 2.063±0.631%, 4.631±1.216%, 2.589±0.75%, and 6.100±1.554%, respectively. Compared to those in the control group, the percentages of CD8^+^ and CD4^+^ T cells in the PDT group and the combined treatment group were significantly increased (Figure 7B, *P*<0.001 or *P*<0.01).

In the MC38 subcutaneous tumor model, the proportions of CD8^+^ TILs in the control group, the PDT group, the BMS202 group and the combined treatment group were 12.207±2.812%, 10.287±2.369%, 10.535±2.426% and 15.712±3.619%, respectively, and the proportions of CD4^+^ TILs were 13.275±3.209%, 14.929±3.591%, 12.066±2.930% and 12.971±3.139%, respectively. Subsequently, we evaluated only the tumor parenchymal region (excluding the tumor stromal region) in the MC38 subcutaneous tumor model. The proportions of CD8^+^ TILs in the control group, the PDT group, the BMS202 group and the combined treatment group were 3.315±0.718%, 8.489±1.955%, 4.904±1.129% and 15.240±3.51%, respectively, and the proportions of CD4^+^ infiltrating lymphocytes were 2.729±0.782%, 6.333±1.608%, 12.799±3.099% and 13.516±3.265%, respectively. There were no significant differences in the percentage of CD8^+^ or CD4^+^ T cells between the TPE-IQ-2O PDT group and the combined treatment group. However, compared to those in the control group, the percentages of CD8^+^ and CD4^+^ T cells in the two groups were significantly increased (Figure 7C, *P*<0.001 or *P*<0.01).

**Figure 8:**
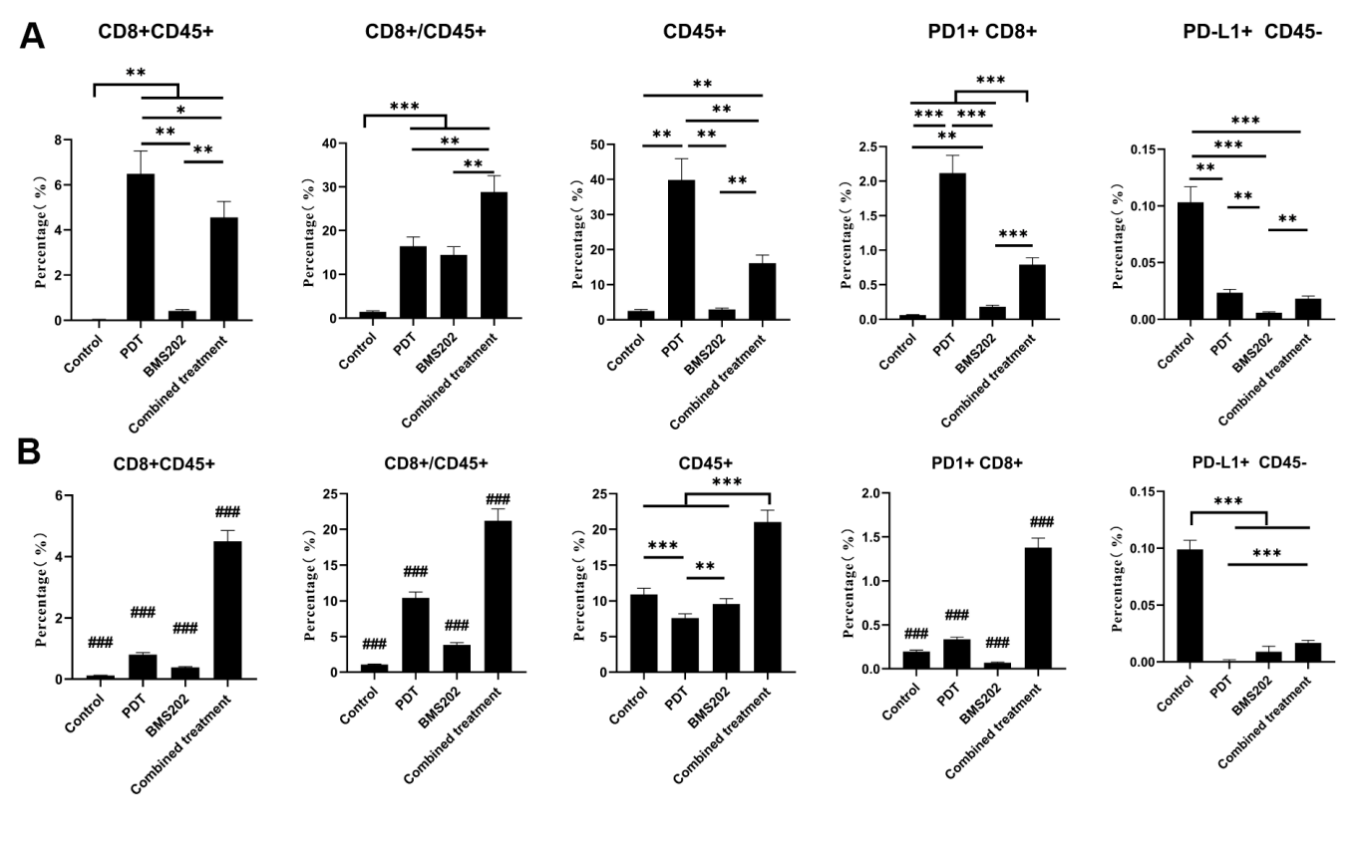
**Flow cytometry showed that TPE-IQ-2O PDT combined with a PD-L1 ICI increased the infiltration of CD8^+^ T cells into tumors.** (**A**) Flow cytometric analysis of a CD8^+^ T lymphocyte-related phenotype in each group of mice bearing a subcutaneous LLC tumor. (**B**) Flow cytometric analysis of the CD8^+^ T lymphocyte-related phenotype in each group of mice bearing a subcutaneous MC38 tumor (*p < 0.05, **p < 0.01, ***and ###p < 0.001 for pairwise comparison following Dunnett’s T3 post hoc test, ### p < 0.001 compared with any other group).

We further analyzed the infiltration of CD8^+^ lymphocytes into LLC and MC38 tumors by flow cytometry, and the proportions of CD8^+^ TILs are shown in Figure 8. In the LLC subcutaneous tumor model, the percentage of CD45^+^, CD8^+^CD45^+^ lymphocytes in the TPE-IQ-2O PDT group and the ratio of CD8^+^/CD45^+^ lymphocytes in the combined group were significantly increased (Figure 8A, *P*<0.001). For MC38 tumors, the proportion of CD45^+^ lymphocytes and the ratio of CD8^+^ PD-1^+^, CD8^+^/CD45^+^ lymphocytes in the combined group were also significantly higher than those in the other groups (Figure 8B, *P*<0.001). The scatter diagram of flow cytometry analysis is shown in Supplementary Figure 1.

## DISCUSSION

Due to their highly metastatic and treatment-refractory nature, malignant tumors are the primary threats to human health worldwide [29, 30]. Therefore, the development of novel treatment strategies for malignant tumors is urgently needed. Our adjuvant TPE-IQ-2O PDT regimen can be combined with either of two treatment modalities, surgery or immunotherapy with a PD-L1 ICI, to achieve a satisfying treatment effect on mouse models. TPE-IQ-2O PDT not only inhibits tumor growth directly via local therapeutic effects but also stimulates systemic antitumor immunity, which further decreases postoperative recurrence and improves the response of PD-L1 ICIs.

PDT research based on AIE materials has developed rapidly over the last few years. As a novel kind of PS, AIE materials exhibit high selectivity and high photostability *in vivo*, subverting the shortcomings of traditional PSs [31, 32]. Among these materials, TPE-IQ-2O is one of the most noteworthy members with potential value for the diagnosis and treatment of cancer. In addition to its function as a PS, TPE-IQ-2O can also serve as a tumor-targeting fluorescent probe. As a charge-dependent material, TPE-IQ-2O was found to be specifically enriched in the mitochondria of tumor cells (Figure 1), which may be due to the elevated mitochondrial membrane potential in tumor cells [33].

The major hurdles to success for most PDTs are tumor specificity, adverse events and the penetration depth of light [34]. Notably, TPE-IQ-2O showed specific staining of both mouse and human tumor cell lines. Subsequently, the targeted killing effect of TPE-IQ-2O PDT on cancer cells was observed *in vitro* (Figure 2). The CCK-8 and Western blot results showed that TPE-IQ-2O PDT had no adverse effect on normal cells. In addition, no obvious damage was found *in vivo* following TPE-IQ-2O PDT treatment. Compared to the traditional 5-ALA PDT group, the TPE-IQ-2O PDT group showed no distant metastasis or skin burning. These results indicate that TPE-IQ-2O PDT is a safe therapeutic approach. However, the antitumor effect of TPE-IQ-2O PDT on mouse models, especially immunodeficient nude mice, was weaker than that found *in vitro* and in a highly transparent zebrafish model. These findings suggest that the limited *in vivo* antitumor effect of TPE-IQ-2O was caused by the weak penetration depth of light. In most PDT methods, insufficient light penetration remains a ‘short plank’ within the deep tumor [35, 36]. To overcome the limitation, one natural idea is to combine PDT with surgery in two steps; namely, perform PDT in the resection margin after reduction.

Prevention of marginal recurrence is crucial in surgery, but there is still no guarantee that a fully cancer-free margin can be achieved in some cases [37, 38]. Currently, surgery combined with local radiotherapy is utilized to reduce the local recurrence of tumors in the clinic with remarkable effects [39–41]. The mechanism of PDT is similar to that of radiotherapy. Based on this similarity, we proposed an adjuvant PDT therapy, namely, surgery combined with TPE-IQ-2O PDT. In this study, prognosis was evaluated in a KYSE-30 cell nude mouse model. There was no obvious effect on the mice in the TPE-IQ-2O PDT alone group, which had a large tumor burden (volume >62.5 mm^3^). TUNEL assays suggested that TPE-IQ-2O PDT could inhibit superficial tumor strongly but not so effective in central tumor cells (Supplementary Figure 8). Namely, there is an upper limit of the tumor load for which the TPE-IQ-2O PDT can provide effective treatment coverage. In addition, increased levels of hypoxia in the microenvironment with tumor progression further decrease the therapeutic efficacies, due to the oxygen dependence of PDT [42]. Therefore, once the tumor growth exceeds a certain threshold, TPE-IQ-2O PDT fails to exert effective influence for its limitation in light energy and oxygen. This result again emphasized that TPE-IQ-2O PDT alone could not effectively eradicate large tumor burdens completely. This finding prompted us to conclude that the therapeutic effect of TPE-IQ-2O PDT was related to the tumor burden. However, a particularly encouraging finding was that no tumor recurrence was observed in the TPE-IQ-2O PDT combined with surgery group in the KYSE-30 and LLC-Luc cell nude mouse models. These results indicate that adjuvant TPE-IQ-2O PDT after surgery can effectively reduce tumor recurrence.

It should be noted that TPE-IQ-2O PDT uses white light as the excitation light. Compared to traditional PDT laser sources, white light is widely accepted as a convenient and safe light source in laparoscopy and gastroscopy. With no additional high-power laser emitter equipment required, TPE-IQ-2O PDT is worthy of clinical application and promotion. Thus, orthotopic implantation tumor models were established to simulate TPE-IQ-2O PDT with a gastroscope. In this model, preimplantation of subcutaneous tumors could improve the success rate of tumor implantation *in situ*, which might be associated with the induction of immune tolerance following subcutaneous tumor implantation. It was more surprising to find that recurrence in the distant subcutaneous incision was reduced with concomitant direct inhibition of the local irradiation site (the orthotopic implanted tumor). We speculate that even in the absence of T cells, TPE-IQ-2O PDT may still inhibit tumors through innate immunity. This is similar to the innate immune response activated by radiotherapy [43, 44].

As a local therapy, PDT is generally observed to improve the immune response via two main mechanisms. First, PDT promotes the release of various inflammatory cytokines and chemokines that enhance antitumor activity [23]. Second, PDT kills tumor cells by apoptosis. Innate immune effector cells phagocytose dying cancer cells and present tumor antigens to T cells, thus inducing a tumor antigen-specific T cell response [45]. We observed significantly elevated CD8^+^ T cell accumulation after TPE-IQ-2O PDT treatment alone, suggesting that this treatment can stimulate adaptive immunity. Additionally, we found that TPE-IQ-2O PDT was more effective in a xenograft model established with normal mice than one established with severely immunodeficient mice. This difference indicates that the immune status, especially the T cell status, can affect the curative effect of TPE-IQ-2O PDT.

Our understanding of tumor immunology has progressed remarkably over the past two decades. At present, immunotherapy, especially PD-1/PD-L1 ICIs that function by inhibiting immune checkpoints and restoring the normal immune system, is the most attractive treatment for cancer. Most of the clinical and animal research on PD-1/PD-L1 ICIs is based on monoclonal antibodies. However, related synthetic small molecule inhibitor research is comparatively limited to date because protein molecules may affect the fluorescence properties of AIE materials. Additionally, compared to monoclonal antibodies, small molecular inhibitors showed beneficial effects in some cases with the advantages of simple synthesis, low cost, stability and low immunogenicity [46]. However, clinical results suggest that PD-1/PD-L1 ICIs seem to be ineffective in patients with a ‘cold’ tumor [47]. To maximize benefits, combining PD-1/PD-L1 ICIs with other treatments will be necessary to enhance the durable response rate of immunotherapy. To cross-link ICIs with TPE-IQ-2O in future studies, we chose to use a chemically synthesized PD-L1 inhibitor, namely, BMS202, rather than antibodies in this study.

To represent the features of ICI-insensitive tumors relatively faithfully, third-generation subcutaneous tumor C57 mouse models were established with LLC or MC38 cells. It should be pointed out that ‘insensitive’ is defined relative to complete remission, especially in MC38 tumor C57 models. The tumors formed by LLC or MC38 cells were designed to imitate immune desert-type and immune excluded-type tumors, respectively. In the above two models, our tumor volume data indicate that TPE-IQ-2O PDT combined with BMS202 could be more effective than BMS202 alone. The immunohistochemistry results paralleled the FACS data. Micrographs of tumor sections showed that more CD8^+^ T cells were recruited to the tumor parenchyma in the TPE-IQ-2O group and combination group than in the BMS202 alone group. These results suggest that TPE-IQ-2O PDT could further activate CD8^+^ T lymphocytes, enhance cellular immunity, and inhibit tumor cell proliferation via synergistic effects produced in combination with BMS202. The relationship between PD-L1 and clinical outcomes remains controversial [48]. Some studies suggest that the efficacy of PD-1/PD-L1-targeted immunotherapy is associated with PD-L1 expression [49]. In our study, the efficacy of BMS202 was different in the two models, despite LLC and MC38 cells having equally low basal levels of PD-L1 expression (<1%) (Figure 8). This indicates that PD-L1 expression may not be the best predictor of response to immunotherapy in these animal models. Previous studies suggest that cancer cells can express many immune checkpoints in addition to PD-L1 to escape immune surveillance [50–52]. It is interesting to note that in the combination groups, the tumors in both models were still not completely eliminated despite the decrease in PD-L1 expression. Thus, we speculate that other immune checkpoints may be involved during combination therapy.

We have clarified the general principle that TPE-IQ-2O PDT can enhance the efficacy of PD-L1 ICI therapy. Another main common denominator of the two models was that the ratio of CD8^+^/CD4^+^ T cells was inverted after TPE-IQ-2O PDT compared with BMS202 alone. Thus, we tentatively propose that increasing the infiltration of CD4^+^ T cells and increasing the infiltration of CD8^+^ T cells are equally important for activating antitumor immunity. In the MC38 C57 subcutaneous tumor model, although TPE-IQ-2O PDT could increase the infiltration of CD8^+^ T lymphocytes, the change was not as obvious as that in the LLC C57 subcutaneous tumor model. In addition, the proportions of CD8^+^CD45^+^ T cells in the total cell population, CD45^+^ cells and PD-1^+^CD8^+^ T lymphocytes increased significantly in the combination group in the MC38 C57 subcutaneous tumor model. This suggests that the mechanisms by which TPE-IQ-2O PDT enhances the response rate in the two models may be different in some points. We thus intend to analyze any differences in the tumor microenvironments in our future work through more analysis of inflammatory cytokines and chemokines. Additionally, we also recognize that the data generated with mouse cancer models are considerably different from those generated in humans.

In summary, TPE-IQ-2O PDT is a novel and safe therapy and we have developed two effective combination therapies based on TPE-IQ-2O PDT: TPE-IQ-2O PDT combined with surgery and TPE-IQ-2O PDT combined with PD-L1 ICIs. TPE-IQ-2O PDT can reduce marginal recurrence after surgery. More importantly, TPE-IQ-2O PDT combined with ICIs can overcome the low response rate of ICIs, promote tumor CD8^+^ T cell infiltration and improve prognosis. Therefore, the combination of TPE-IQ-2O PDT with ICI therapy or surgery described in our research provides a promising strategy for cancer therapy.

## MATERIALS AND METHODS

### *In vitro* experiments

### Cell culture and TPE-IQ-2O solution preparation

BEAS-2B and LO-2 cells were cultured in Bronchial Epithelial Growth Medium (BEGM; East Rutherford, USA) and Dulbecco’s modified Eagle’s medium (DMEM)/F12 (Gibco, USA), respectively. DMEM (Gibco, USA) was used to culture MCF-7, A549, MC38, HepG2, HCC827, Lewis lung carcinoma (LLC) cells, 3T3, 293T, and human umbilical vein endothelial cells (HUVECs). RPMI-1640 (Gibco, USA) medium was used to culture KYSE-180, NCI-H1299, NCI-H358, HCC827, KYSE-30, KYSE-450 and NCI-H510 cells. MC38 cells were purchased from the China Center for Type Culture Collection (CCTC, Beijing, China). KYSE-30, KYSE-450, KYSE-30 and LO-2 cells were obtained from the cell bank of the Chinese Academy of Sciences (CAS, Shanghai, China). Other cells were purchased from the American Type Culture Collection (ATCC). All the cells were cultured in medium supplemented with 10% fetal bovine serum (FBS; Gibco) at 37°C in 5% CO2.The details of cell lines are summarized in Supplementary Table 1.

TPE-IQ-2O was purchased from AIEgen Biotech Co., Ltd. TPE-IQ-2O (0/100/200/400/800 nM) was prepared by adding 0/4/8/16/32 μL of a 0.1 mM stock solution of TPE-IQ-2O in dimethyl sulfoxide (DMSO; Thermo Fisher, USA) to 4 mL of culture medium.

### TPE-IQ-2O localization observation

MitoTracker Deep Red (MTDR), a special dye targeted to mitochondria, was purchased from Invitrogen. A549 cells were stained simultaneously with MTDR (50 nM) and TPE-IQ-2O (200 nM) to assess the localization of TPE-IQ-2O. After 25 minutes of incubation, the cells were washed, and fresh phenol red-free medium was added. Ultra-high-resolution fluorescence microscopy (DeltaVisionTM OMX SR, GE, USA) was used to capture images and verify the colocalization of mitochondria and TPE-IQ-2O.

### Quantitating the fluorescence intensity of TPE-IQ-2O staining

After 24 h of cell culture, the cell density of each plate was approximately 80%. Cancer cells (MCF-7, A549, MC38, HepG2, HCC827, LLC, KYSE-180, NCI-H1299, NCI-H358, HCC827, KYSE30, KYSE-450 or NCI-H510 cells) and normal cells (3T3, 293T, HUVEC, BEAS-2B or LO-2 cells) were incubated with different concentrations of TPE-IQ-2O (0/100/200/400/800 nM) in 96-well plates for 25 minutes. A multifunctional fluorescent enzyme label instrument (Varioskan LUX, Thermo Fisher, USA) was used to read and analyze the fluorescence intensity (λex: 430 nm, λem: 560 nm). The average and standard deviation of the cellular fluorescence intensities of each well were calculated with a self-contained software system (SkanIt Software 4.1 for Microplate Readers RE, ver. 4.1.0.43, Thermo Fisher).

### Cytotoxicity evaluation

Cancerous LLC and A549 cells and normal BEAS-2B cells were incubated with different concentrations of TPE-IQ-2O to evaluate cytotoxicity. According to the method for Cell Counting Kit-8 (CCK-8; Beyotime, C0039, China) assays, the concentrations of A549 and BEAS-2B cells in 96-well plates were 3000 cells/100 μL per well. After 24 h of incubation, different concentrations of TPE-IQ-2O (0/100/200/400/800 nM) were added to the plate for 25 minutes. Two plates containing A549 and BEAS-2B cells were exposed to white light (5 W, 85 mW/cm^2^) for 20 minutes, and another two plates remained in the dark as controls. Then, the medium in each well was replaced with a CCK-8 solution. After 2 h of incubation, cytotoxicity was determined by measuring the absorbance of the 96-well plate wells at 450 nm with a microplate reader.

### Measurement of the mitochondrial membrane potential

The mitochondria membrane potential was determined by JC-1 kit (Beyotime, C2006, China). Fluorescent images of JC-1 were taken on a Olympus IX73 microscope using green (λ_ex_: 482/18 nm, λ_em_: 520/28 nm) and red channels (λ_ex_: 560/14 nm, λ_em_:605/52 nm). JC-1 quantification was measured by BD LSR II flow cytometry using FITC (green) (λ_ex_: 488 nm, λ_em_: 530/30 nm) and PE (red) (λ_ex_: 488 nm, λ_em_:575/26 nm) channels.

### Measurement of ROS levels

ROS production was investigated by 2 μM Dihydroethidium (DHE, Beyotime, S0063, China). ROS levels was measured by BD LSR II flow cytometry using PE (λ_ex_: 488 nm, λ_em_:575/26 nm) channels.

### *In vivo* experiments

### Cell lines and animals

Luciferase-tagged LLC cells (LLC-Luc cells) were transfected with the firefly luciferase (Luc) reporter gene using a lentivirus as described previously [53]. mCherry-tagged A549 cells (mCherry^+^ A549 cells) were obtained by transfection of the mCherry reporter gene using lentiviral vectors as described previously [54]. LLC-Luc and mCherry^+^ A549 cells stably expressed the corresponding reporter gene. LLC-Luc cells, KYSE-30 cells, and MC38 cells were used to establish tumor-bearing mouse models. mCherry^+^ A549 cells were used to establish a zebrafish xenograft model.

Female C57BL/6 mice and BALB/c nude mice aged 6-8 weeks were purchased from Beijing Huafukang Biological Co., Ltd. Adult zebrafish (AB) were obtained from the Model Animal Research Center of Nanjing University. All the experimental animals were raised in the Experimental Animal Center of the Chinese Academy of Medical Sciences. Mice were maintained in a specific-pathogen free (SPF) environment. All animal experiments were conducted in accordance with animal protection guidelines and were approved by the Committee of the Cancer Hospital of the Chinese Academy of Medical Sciences. To minimize animal pain and protect animal welfare, all operations on mice were performed under inhaled isoflurane anesthesia. Tumor growth and response to therapy were monitored by caliper measurements, and tumor volume was calculated using the formula a^2^×b×0.5, with a being the short dimension and b being the long dimension. Mice were euthanized for ethical reasons if their tumor became larger than 2,000 mm^3^. For a more comprehensive summary information about these animal models, see Supplementary Table 2.

### Zebrafish xenograft assay

To observe treatment effects eliminating the limitations related to the penetration depth of light, zebrafish embryos generated from parent adult fish were used. Briefly, mCherry^+^ A549 cells were transplanted into zebrafish embryos as follows: 0.0003% tricaine (Sigma-Aldrich, St. Louis, MO, USA) was used as an anesthetic, and 100-200 mCherry^+^ A549 cells/fish were injected into the yolk sac of zebrafish embryos 48 h after insemination. For drug delivery by soaking, the zebrafish embryos were randomly transferred to 24-well plates (10 embryos/well) at 2 days post injection. Each well of embryo medium contained 200 nM TPE-IQ-2O and received 15 minutes of 5 W white light (85 mW/cm^2^) treatment or no light treatment. Then, we observed the fish *in vivo* by fluorescence microscopy (SMZ18, Nikon, Japan). The detailed methods for microinjection and feeding were previously described [55].

### 5-Aminolevulinic acid (5-ALA) PDT contrast test

5-ALA was purchased from Shanghai Fudan Zhangjiang Bio-Pharmaceutical Co., Ltd., China. 118 mg 5-ALA was dissolved in 0.5 ml PBS and then the solution (100 μL/mouse) was administered by peritumoral injection in 5-ALA PDT group(n=5). Three hours later, the tumor area was irradiated using a laser of wavelength 635 nm for a duration of 15 min. the TPE-IQ-2O PDT group (n=5) was administered 100 μL of 800 nM TPE-IQ-2O (containing 0.8% DMSO) by peritumoral injection and subjected to white light exposure (5 W, 85 mW/cm^2^) for a duration of 15 min. The control group (n=5) was administered by 100 μL of PBS (containing 0.8% DMSO) only. The LLC-Luc subcutaneous model is described in the following paragraphs.

### Subcutaneous lung and esophageal xenograft models and an orthotopic esophageal cancer model established with BALB/c nude mice

Briefly, xenograft models were established by subcutaneous injection of 7×10^6^ cells/ml (200 μL) LLC-Luc or KYSE-30 cells into the right hind leg.

A KYSE-30 cell suspension with a concentration of 7×10^6^ cells/ml (50 μL) was injected into the esophagus to establish an orthotopic esophageal cancer model. The PDT method for treating subcutaneous tumors was the same as that used for treating C57BL/6 mice. For orthotopic esophageal cancer, 100 μL of 800 nM photosensitizer was slowly injected along the esophagus with a gastric perfusion needle, and an optical fiber was inserted into the esophagus through the gastric perfusion needle. Light was introduced into the esophagus through the optical fiber.

### Establishment of a relatively ICI-insensitive subcutaneous implant model in C57BL/6 mice

In preparation for the development of a stable subcutaneous tumor model with low sensitivity to PD-1/PD-L1 inhibitors, 1×10^6^ cells/mL MC38 or LLC-Luc cells were first implanted into five C57BL/6 mice. The mice were then treated every two days with intraperitoneal injections of the ICI BMS202 (200 μL, 200 nM) (Selleck, S791202, China). The mice with the fastest tumor growth were selected. The tumor mass was dissected under isoflurane gas anesthesia and placed in Hanks’ buffer to remove any blood cells and separate the necrotic tissue. The tumor tissue was cut into approximately 2×2×2 mm^3^ pieces. Then, tumor pieces were implanted subcutaneously via a cannula needle into new mice for passage *in vivo*. Subsequently, the tumor masses of 1-2 mice with rapid tumor proliferation were selected as insensitive tumor masses and implanted into a new batch of mice for the experiment.

Mice were divided into four groups (5 mice per group): the control group, the PDT group, the BMS202 group and the combined treatment group. The control group was locally injected with 100 μL of 0.8% DMSO around the tumor, the light group was irradiated with a 5 W LED white light (85 mW/cm^2^) source, and the TPE-IQ-2O group was locally injected with 100 μL of 800 nM TPE-IQ-2O (containing 0.8% DMSO) around the base of the tumor. In the PDT group, the tumor was irradiated with white light after injection of TPE-IQ-2O. The BMS202 group was intraperitoneally injected with 200 μL of BMS202 (200 nM), and the combined treatment group was treated with TPE-IQ-2O PDT combined with BMS202.

### Imaging observation

An IVIS Xenogen spectrum imager (PerkinElmer, Waltham, MA, USA) was used for *in vivo* imaging. Animals were intraperitoneally injected with fluorescein (150 mg/kg; Shanghai Bi-Yun-Tian Biotechnology, Shanghai, China) 5 minutes before analysis. The animals were then anesthetized with an isoflurane carburetor. The rectangular region of interest (ROI) was evaluated and analyzed with Living Image software v.4.2 (PerkinElmer).

### Two-photon 3D imaging of tumor vessels

Two-photon images were acquired using an LSM 880 NLO (Zeiss, Germany) at room temperature. NIR805 (AIEgen Biotech Co., Ltd., China) was used as a blood vessel imaging dye and shows maximal emission at 805 nm. NIR805 (1 mg) was dissolved in 1 ml of DMSO solution as the working fluid. Then, 3 μl of NIR805 solution was added to 97 μl of PBS to make a 3% NIR805 dye solution immediately before use. Next, 100 μL of 3% NIR805 dye was injected into mice via the tail vein. Following 15 minutes of staining, images were collected by increasing the imaging depth to 800 μm. A femtosecond (fs) laser beam with a wavelength of 1040 nm was used as the excitation source. Second-harmonic generation (SHG) signals at the wavelengths of 575-610 nm and 805 nm were collected to observe the stroma and blood vessels, respectively.

### Western blot analysis

Western blotting was performed with the following antibodies: anti-cleaved caspase-3 (Cell Signaling Technology, 1:1000 dilution), anti-Bcl-2 (Cell Signaling Technology, 1:1000 dilution), anti-Bax (Cell Signaling Technology, 1:1000 dilution), and anti-GAPDH (Cell Signaling Technology, 1: 1000 dilution). Rapid gel kits were prepared using NewFlash Protein AnyKD PAGE (BioSci) for cell protein samples.

### Hematoxylin and eosin (HE) staining, TUNEL staining and immunohistochemistry (IHC)

Tissue specimens were fixed with 4% paraformaldehyde for 24 h and then embedded in paraffin. Five-micron-thick sections were cut from the formalin-fixed, paraffin-embedded (FFPE) tissue masses. For HE staining, the sections were subjected to HE staining as described previously [56]. For TUNEL staining, the sections were performed as previously described using *In Situ* Cell Death Detection Kit (Roche, 11684795910, USA) [57].

For IHC, sections were baked at 85° C for 1 h and then treated with xylene for deparaffinization and gradient alcohol for hydration. High pressure and a pH=6 citrate buffer was used for 2 minutes for antigen repair. After the blockade of endogenous peroxidase activity with 3% hydrogen peroxide, the sections were blocked with 10% goat serum, followed by incubation with an anti-CD8 antibody and anti-CD4 antibody (Abcam) overnight. The next day, after washing with TBST, the sections were incubated with an HRP-coupled secondary antibody for 30 minutes at room temperature. Color development was performed by using a DAB kit (BOSTER Biological Technology Co., Ltd., Cat No. AR1021, China).

### Flow cytometry

Flow cytometry analysis was performed according to a protocol described previously using a FACS LSRII (BD Biosciences, NJ, USA) [58]. Briefly, tumor tissue samples were digested with collagenase and trypsin into single cells. After termination of the digestion, the cells were collected and washed via centrifugation, followed by removal of erythrocytes using Red Blood Cell Lysing Buffer (Sigma-Aldrich). All cells were stained with an antibody cocktail (anti-PD-L1 APC, Cat No. 564715; anti-CD8 PE, Cat No. 553033; anti-CD45 FITC, Cat No. 553079; all BD Biosciences, and anti-PD-1 PE-Cy7, Cat No.25-9985-82, eBioscience). Staining was compared with isotype control antibody (BD Biosciences) staining to correct for nonspecific binding.

### Statistical analysis

Data are shown as the mean ± standard deviation obtained from at least three independent parallel trials. Statistical significance was determined with Fisher’s exact Chi-square test, unpaired Student’s t-test, the least significant difference (LSD) test, the Dunnett test or Dunnett’s T3 post hoc test using SPSS software version 19.0. A p value less than 0.05 was considered significant.

## Supplementary Material

Supplementary Figures

Supplementary Tables
