# Supplementary material for: The therapeutic significance of the novel photodynamic material TPE-IQ-2O in tumors
Source: Aging (Albany NY). 2020 Dec 19;13(1):1383–409. doi: 10.18632/aging.202355 (PMC7835032; doi:10.18632/aging.202355)
Supplement: Supplementary Figures [file aging-13-202355-s001.pdf]

SUPPLEMENTARY FIGURES

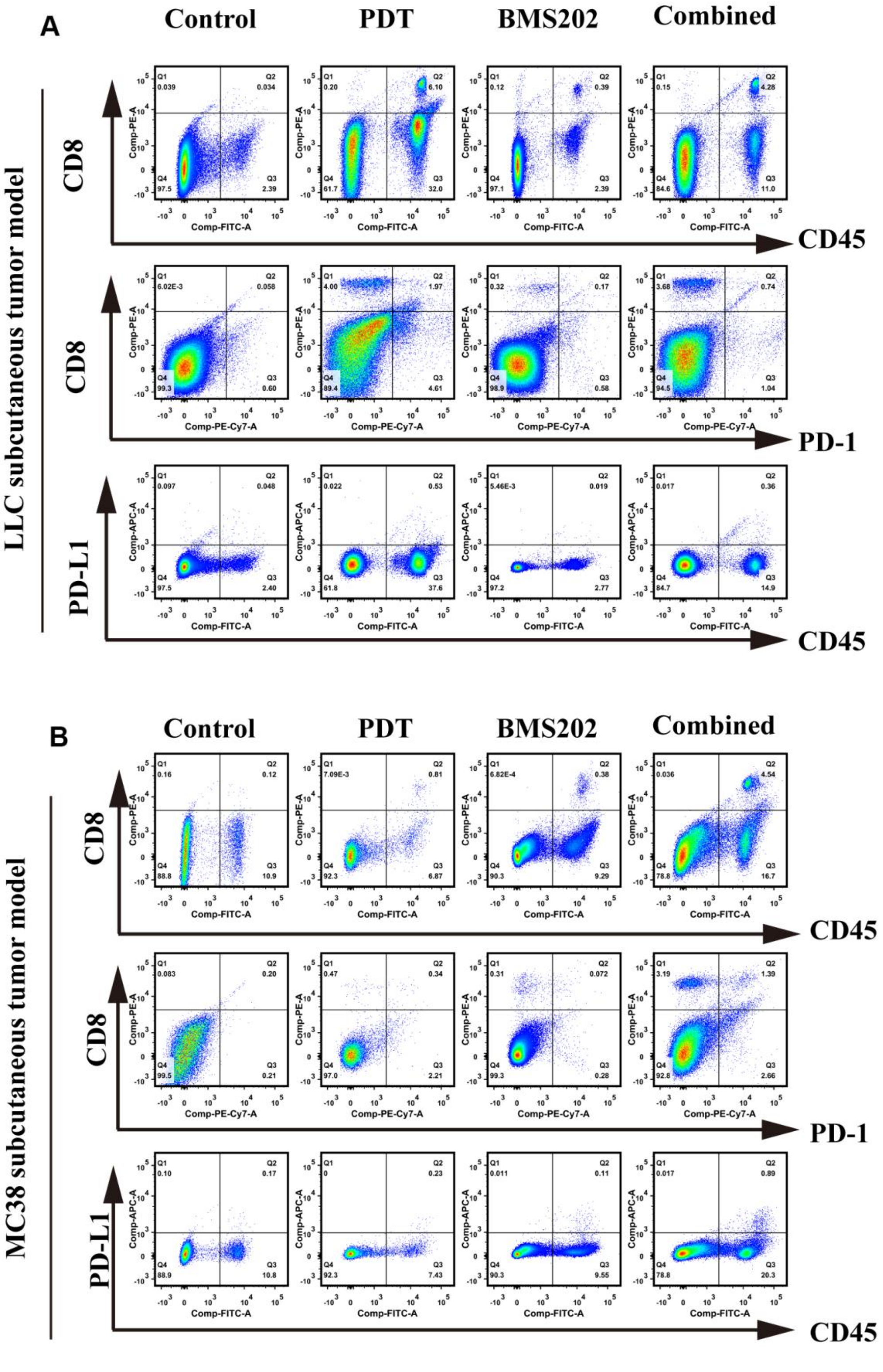

Supplementary Figure 1. Scatter diagram of flow cytometry analysis in each group in LLC subcutaneous tumor model (**A**) and MC38 subcutaneous tumor model (**B**).

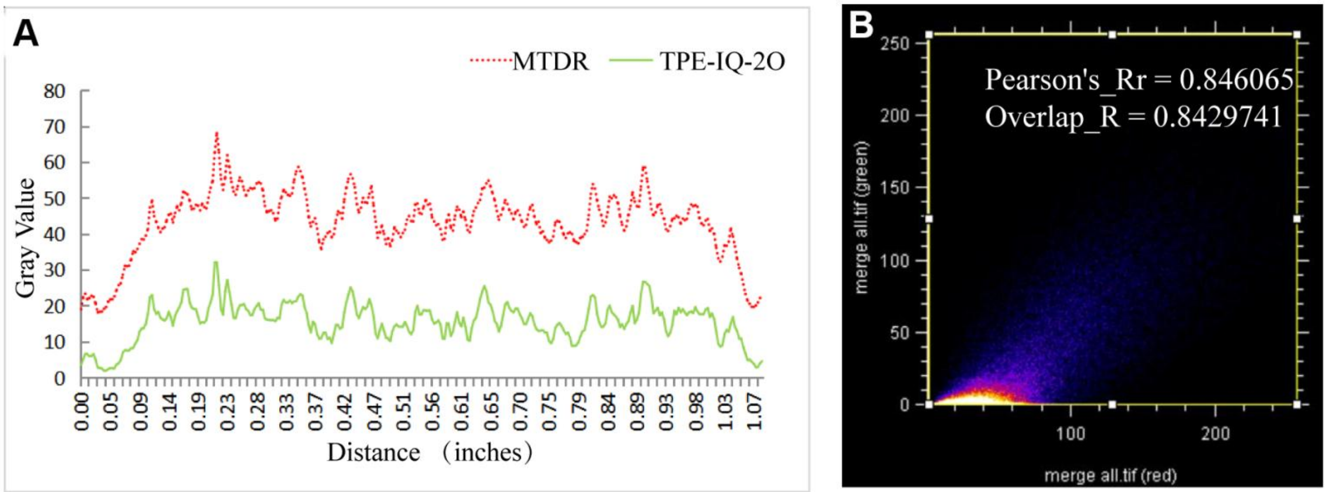

Supplementary Figure 2. The staining coefficient of TPE-IQ-2O. (**A**)The gray value of MTDR and TPE-IQ-2O were calculated using the Image J Plot Profile plugin. (**B**) The Mander's overlap coefficient (R = 0.8429741) and Pearson's correlation coefficient (Rr = 0.846065) were determined using the Image J co-localization plugin.

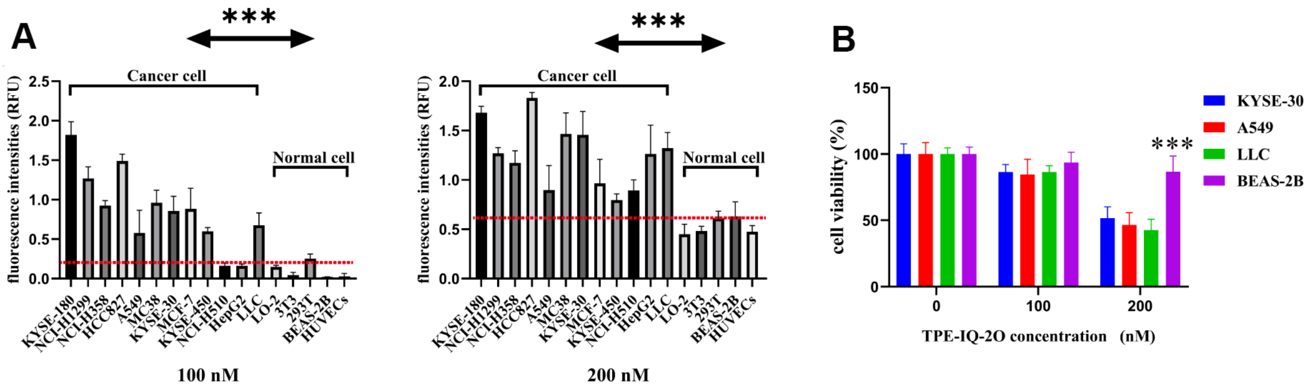

Supplementary Figure 3. Selecting optimal concentration for TPE-IQ-2O. (**A**) The fluorescence intensities of cells incubated under the concentrations of 100 nM and 200 nM; λ_{ex}: 430 nm, λ_{em}: 560 nm, the red dotted line indicates the highest average fluorescence intensities of normal cell lines, ****P*<0.001 by unpaired Student's t-test. (**B**) Cell viabilities measured by CCK8 assay, ****P*<0.001 vs all other cell lines at 200 nM.

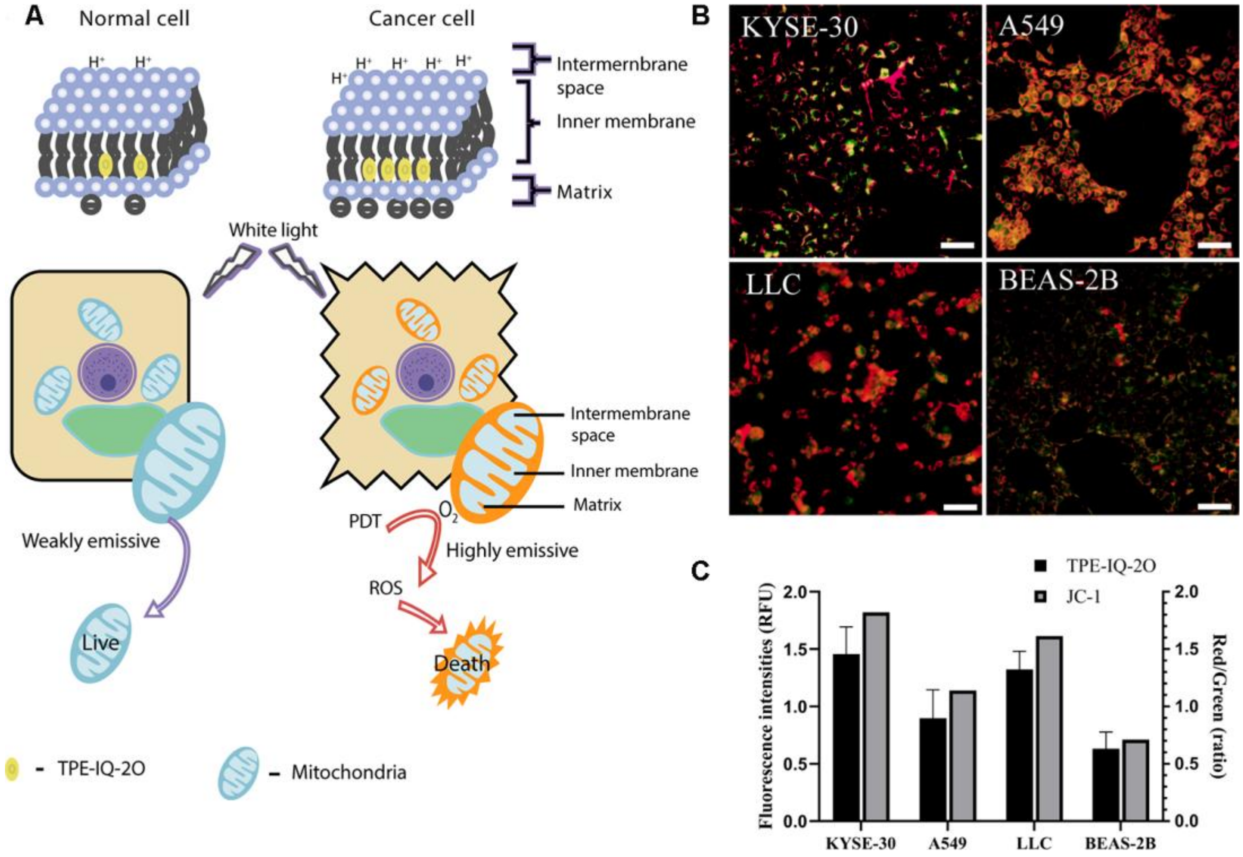

Supplementary Figure 4. Tumor targeting mechanism of TPE-IQ-2O. (**A**) Schematic illustration of TPE-IQ-2O as tumor-targeting PS (Modified from [26]). (**B**) Representative images of JC-1 (2 μM) staining (JC-1 monomer and JC-1 aggregate exhibited green and red fluorescence, respectively, scar bar = 50 μm). (**C**) TPE-IQ-2O and JC-1 staining was analyzed by flow cytometry. (Black) Fluorescence intensity of different cells stained with TPE-IQ-2O (200 nM) for 20 min at FITC channel and (Grey) Mitochondrial membrane potential of different cells was assessed with JC-1 (2 μM) at PE (red) channel and FITC (green) channel (red/green ratio was represented as the ratio of aggregated and monomeric JC-1.

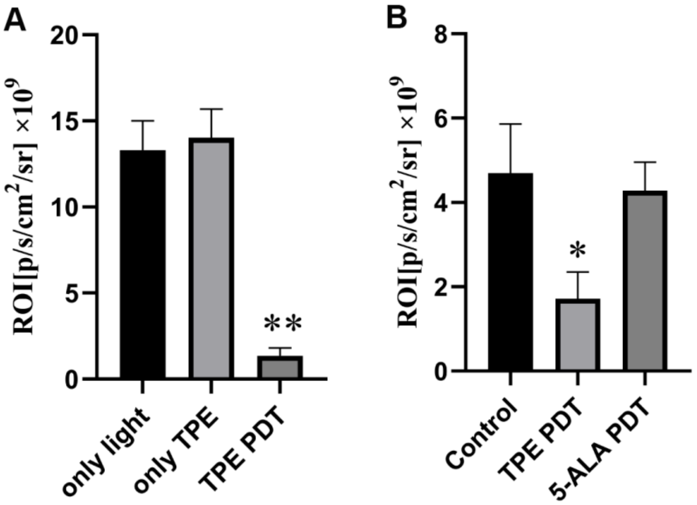

Supplementary Figure 5. Quantitative analysis of tumor imaging in vivo in only light, only TPE-IQ-2O, TPE-IQ-2O PDT group (**A**) and different PDT groups (**B**)**P*<0.05, ***P*<0.01 vs all other groups).

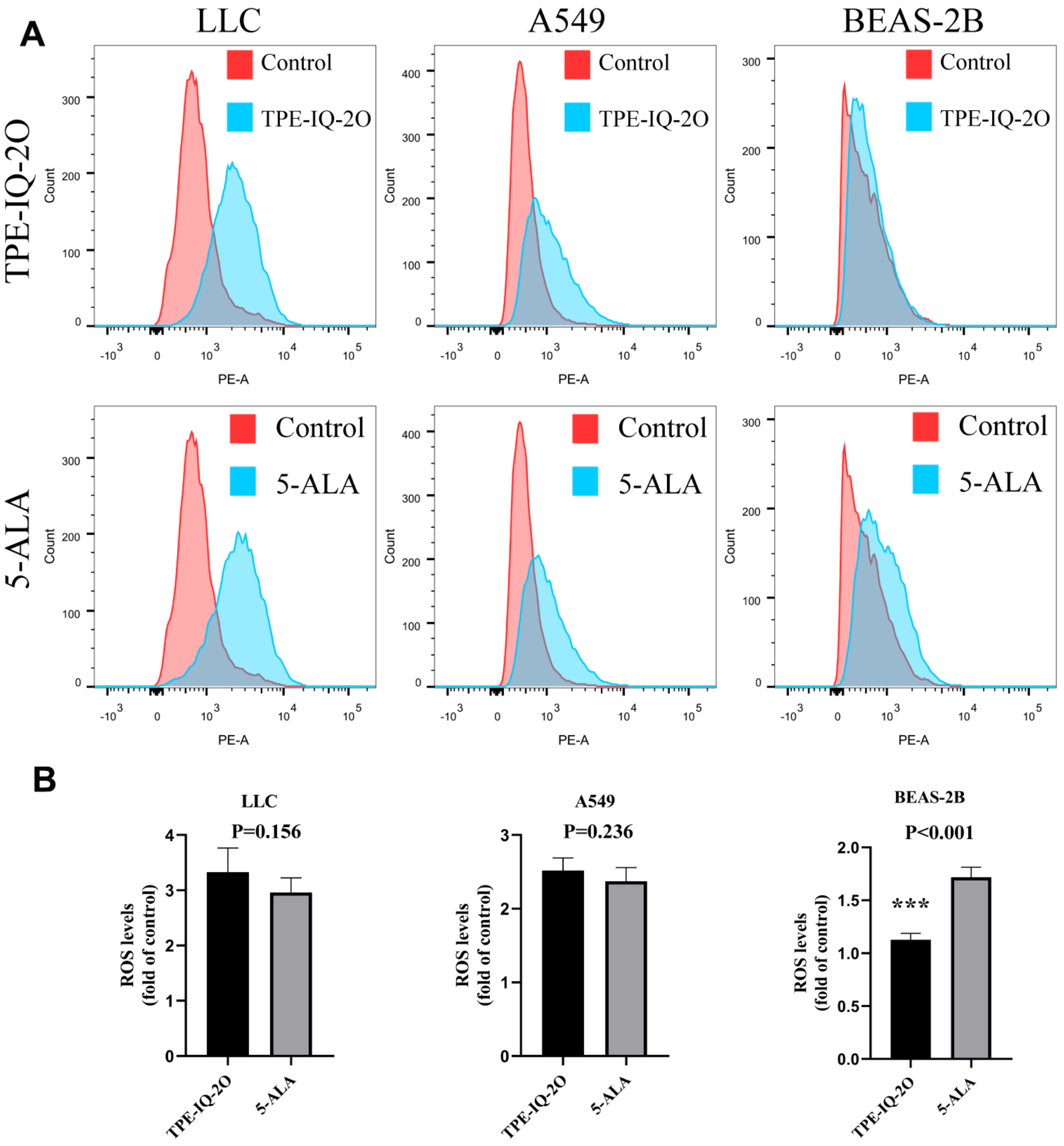

Supplementary Figure 6. Comparative analysis of TPE-IQ-2O and 5-ALA to induce ROS in different cells. (**A**) Representative flow cytometry plots of DHE in different groups. (**B**) Histogram shows quantitative analysis of results of flow cytometry, ****P*<0.001 vs 5-ALA.

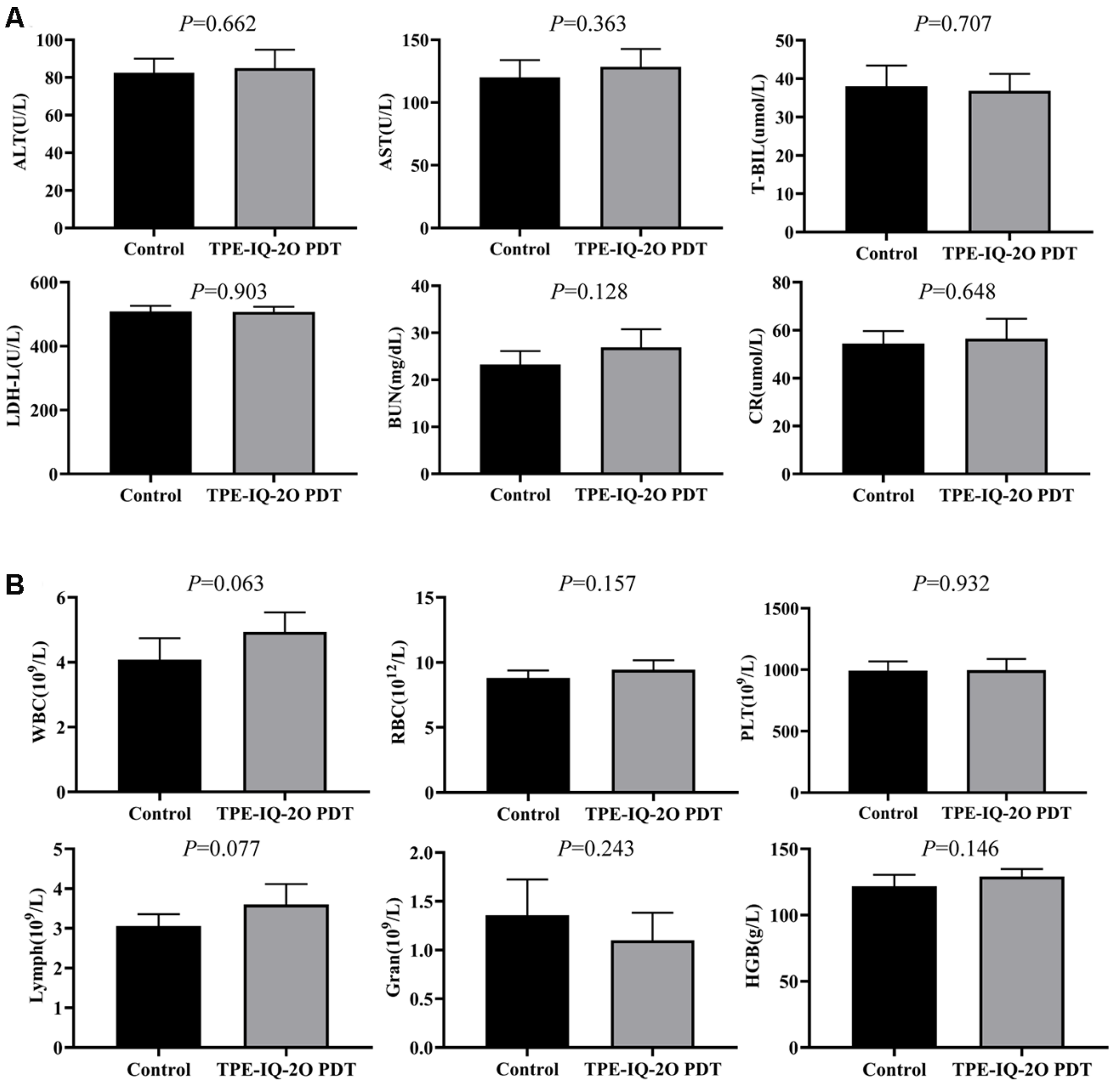

Supplementary Figure 7. Comparison of biochemical (**A**) and blood cell (**B**) analysis in control and TPE-IQ-2O PDT group (The blood of each group was detected by Chemray 800 biochemical analyzer and BC-2800vet blood cell analyzer).

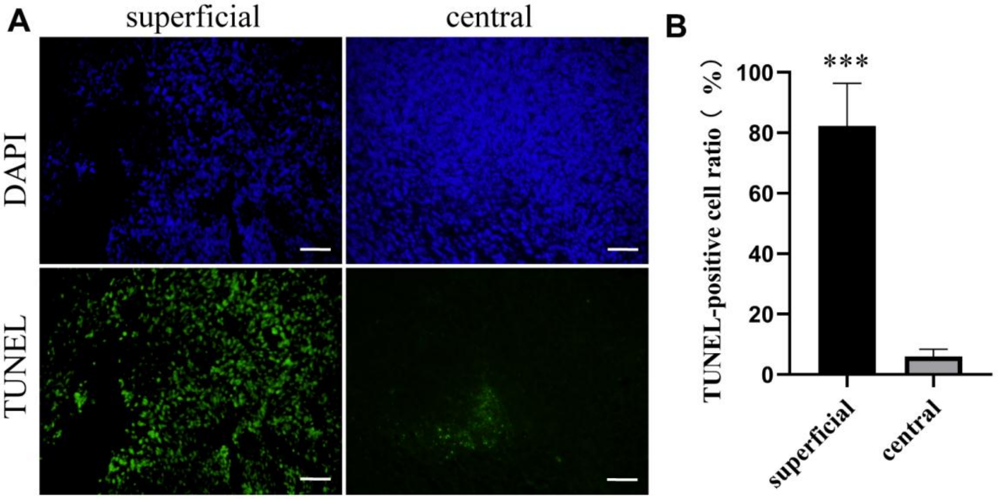

Supplementary Figure 8. TUNEL staining images (**A**) and quantification analysis by Image J (**B**) of the superficial and central LLC tumor (Green indicates the TUNEL-positive cells, Scar bar=50 μm, ****P*<0.001 vs central tumors).
