# Supplementary material for: The therapeutic significance of the novel photodynamic material TPE-IQ-2O in tumors
Source: Aging (Albany NY). 2020 Dec 19;13(1):1383–409. doi: 10.18632/aging.202355 (PMC7835032; doi:10.18632/aging.202355)
Supplement: Supplementary Tables [file aging-13-202355-s002.pdf]

SUPPLEMENTARY TABLES

Supplementary Table 1. Basic information of cell lines.

Cell line	Cell type	Species	Tissue	Culture medium	Source
LLC	lung cancer cell	mouse	lung	DMEM	ATCC
A549	lung cancer cell	human	lung	DMEM	ATCC
HCC827	lung cancer cell	human	lung	DMEM	ATCC
NCI-H1299	lung cancer cell	human	lung	RPMI-1640	ATCC
NCI-H358	lung cancer cell	human	lung	RPMI-1640	ATCC
NCI-H510	lung cancer cell	human	lung	RPMI-1640	ATCC
KYSE-180	esophageal squamous cell carcinoma cell	human	esophagus	RPMI-1640	CAS, Shanghai, China
KYSE-30	esophageal squamous cell carcinoma cell	human	esophagus	RPMI-1640	CAS, Shanghai, China
KYSE-450	esophageal squamous cell carcinoma cell	human	esophagus	RPMI-1640	CAS, Shanghai, China
HepG2	human hepatocarcinoma cell	human	liver	DMEM	ATCC
MCF-7	breast adenocarcinoma cell	human	breast	DMEM	ATCC
MC38	colon carcinoma cell	mouse	colon	DMEM	CCTCC, Beijing, China
BEAS-2B	normal bronchial epithelial cell	human	lung	BEGM	ATCC
LO-2	normal liver cell	human	liver	DMEM/F12	CAS, Shanghai, China
3T3	mouse embryo fibroblast	mouse	embryo	DMEM	ATCC
293T	human kidney epithelial cell	human	kidney	DMEM	ATCC
HUVEC	human umbilical vein endothelial cells	human	vein	DMEM	ATCC

Supplementary Table 2. Basic information of animal model construction.

Animal(Source)	Model	Implants	Main treatment
AB zebrafish (Model Animal Research Center of Nanjing University)			
	zebrafish xenograft mode	mCherry^{+} A549 cells	TPE-IQ-2O PDT
Babl/c nude mice (Beijing Huafukang Biological Co., Ltd)			
	Subcutaneous lung xenograft model	LLC-Luc cells	TPE-IQ-2O PDT, Surgery+TPE-IQ-2O PDT
	Subcutaneous esophageal xenograft model	KYSE-30 cells	Surgery+TPE-IQ-2O PDT
	orthotopic esophageal cancer model	KYSE-30 cells	Surgery+TPE-IQ-2O PDT
C57BL/6 mice (Beijing Huafukang Biological Co., Ltd)			
	LLC tumor-bearing mice	LLC cells	BMS202+TPE-IQ-2O PDT
	MC38 tumor-bearing mice	MC38 cells	BMS202+TPE-IQ-2O PDT
